# Surface landmark quantification of embryonic mouse craniofacial morphogenesis

**DOI:** 10.1186/1471-213X-14-31

**Published:** 2014-07-24

**Authors:** Christopher J Percival, Rebecca Green, Ralph Marcucio, Benedikt Hallgrímsson

**Affiliations:** 1 Department of Cell Biology and Anatomy, Alberta Children’s Hospital Institute for Child and Maternal Health, The McCaig Bone and Joint Institute, University of Calgary, Calgary, AB T2N 4N1, Canada; 2 Department of Craniofacial Biology and Program in Reproductive Sciences, University of Colorado – Denver, Denver, CO 80045, USA; 3 Department of Orthopaedic Surgery, The Orthopaedic Trauma Institute, UCSF School of Medicine, San Francisco, CA 94143, USA

**Keywords:** Morphometrics, Landmark error, Facial prominences, Craniofacial morphogenesis, Mouse embryo, 3D imaging, Micro-CT

## Abstract

**Background:**

Morphometric quantification of subtle craniofacial variation in studies of experimentally modified embryonic mice has proved valuable in determining the effects of developmental perturbations on craniofacial morphogenesis. The direct comparison of landmark coordinate data from embryos of many different mouse strains and mouse models can advance our understanding of the bases for craniofacial variation. We propose a standard set of craniofacial surface landmarks, for use with embryonic day (E) 10.5-12.5 mice, to serve as the foundation for this type of data compilation and analysis. We quantify the intra- and inter-observer landmark placement variation associated with each landmark and determine how the results of a simple ontogenetic analysis might be influenced by selection of landmark set.

**Results:**

Intraobserver landmark placement error for experienced landmarkers generally remains below 0.1 mm, with some landmarks exhibiting higher values at E11.5 and E12.5. Interobserver error tends to increase with embryonic age and those landmarks defined on wide inflections of curves or facial processes exhibit the highest error. Landmarks with highest intra- or inter-observer are identified and we determine that their removal from the dataset does not significantly change the vectors of craniofacial shape change associated with an ontogenetic regression.

**Conclusions:**

Our quantification of landmark placement error demonstrates that it is preferable for a single observer to identify all landmark coordinates within a single study and that significant training and experience are necessary before a landmarker can produce data for use in larger meta-analyses. However, we are confident that this standard landmark set, once landmarks with higher error are removed, can serve as a foundation for a comparative dataset of facial morphogenesis across various mouse populations to help identify the developmental bases for phenotypic variation in the craniofacial complex.

## Background

Morphometric quantification of variation in complex phenotypes is increasingly important to developmental studies of morphogenesis [1-3]. Integrating morphometric methods into studies of experimentally modified embryonic development has proved valuable in determining the simultaneous effects of a given perturbation on morphogenesis across the developing head (eg. [4-9]). Similarly, these methods allow quantification of subtle changes in phenotype that is necessary when examining the simultaneous effects of multiple factors on development of a given trait, as is increasingly common in systems-biology informed approaches [3].

Landmark based morphometric methods have commonly been used to quantify the size and shape of individual craniofacial bones, a skeletal region, or the whole skull (eg. [6,10-15]). While skeletal landmarks have been used to quantify craniofacial morphogenesis during the late embryonic period, there is also a need to quantify the effects of epithelial-mesenchymal interactions that control initial growth and fusion of facial prominences before ossification begins and which have a significant influence on subsequent craniofacial morphology [16-19]. Given a lack of skeletal features and difficulties distinguishing soft tissue layers using computed tomography and other 3D imaging modalities during the earliest period of facial morphogenesis, the external ectodermal surface of the embryo provides the best features upon which to place landmarks for the measurement of craniofacial form [3].

Head surface landmarks based on either 2D photographs or 3D surfaces have previously served as a basis for quantifying the effect of developmental perturbations in embryonic chicks [7,20-22] and mice [5,6,23-25]. With increasing demand for quantification of craniofacial morphogenesis during the earlier embryonic period [26], the direct comparison of morphometric data between studies of different species, mouse strains, disease models, and ages becomes an enticing possibility. Just as large collections of publically-available and well-annotated genomic data facilitate new directions in hypothesis-driven research, a phenomic collection of directly comparable morphometric data has the potential to advance our understanding of the bases for typical and dysmorphic craniofacial variation [26,27].

**Figure 1:**
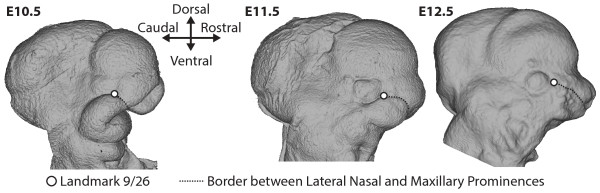
**Surface craniofacial morphology of E10.5-E12.5 mouse embryos.** Right lateral view of the surface craniofacial morphology of E10.5, E11.5, and E12.5 reference specimens in standard orientation, with definitions of the anatomical directions used in the landmark definitions. The location of landmark 9/26 (circle) is noted at the caudal-lateral end of the border between the lateral nasal prominence (dorsal of dashed line) and the maxillary prominence (ventral of dashed line).

Standardization of landmark definitions and confidence that datasets produced by multiple observers are comparable is necessary before morphometric analyses of landmarks from across a large number of mouse populations can be completed. Typical difficulties encountered when defining good landmarks (as discussed in [28-30]) are exacerbated for ectodermal embryonic surfaces because the rapid growth of facial prominences and associated shape changes make it more difficult to define homologous landmarks (Figure 1). In addition, less reliable landmarks may be necessary to adequately quantify phenotypic traits across the head [3,25]. Keeping these difficulties in mind, we defined and tested a standard set of landmarks on the exterior surface of embryonic mice that include only explicitly homologous landmarks to serve as the basis for ontogenetic analyses of facial prominence growth and development between embryonic days (E)10.5, E11.5, and E12.5.

A biological definition of each landmark (Table 1), practical definitions of landmark placement, illustrations defining standard orientation and anatomical directions, and examples of landmarks placed on the 3D surface of a reference specimen at each age were developed in an attempt to maximize consistency in landmark placement by and between observers Additional files 1, 2, 3, 4 and 5. Explicit biological definitions facilitate the interpretation of results from morphometric analyses by tying landmarks directly to biological features of interest. The practical definitions, which can vary by age, were written to guide the placement of each landmark so that it matches the biological definition. The illustrations and landmark placement examples were meant to take the guesswork out of interpreting written definitions. Four observers, three of whom had experience taking landmarks and one of whom had never landmarked before, used these resources to collect landmark coordinates from a sample of E10.5, E11.5, and E12.5 mouse embryos in order to measure the intra- and inter-observer error associated with this landmark set.

**Table 1:** Biological definitions of all landmarks and landmark subset categories

**LM #**	**Abbreviated biological definition**	**Landmark subsets**	**Trouble landmarks**
**1**	The dorsal midline point between the growing forebrain and midbrain lobes.	Non-Facial	
**2**	The dorso-rostral midline extent of the growing forebrain.	Non-Facial	Trouble
**3**	The midline dorsal most extent of the face.	Nasal	
**4**	Midline most rostral extent of the medial nasal processes.	Nasal	
**5**	The ventral midline point on the primary palate, in the region that develops into the vermillion of the lip.	Nasal	
**6 (23)**	The border between the medial nasal process and the forebrain, in line with the center of the body of the medial nasal process.	Nasal	
**7 (24)**	Most dorsal extent of the rostral portion of the lateral nasal process, marking the original rostrolateral intersection of the lateral nasal process and the forbrain.	Nasal	Trouble
**8 (25)**	The dorso-caudal most point of the lateral nasal process.	Non-Facial	
**9 (26)**	The caudal most point of the intersection between the lateral nasal process and the maxillary process, representing the caudal end of the future nasolacrimal duct.	Nasal	
**10 (27)**	The caudo lateral projection of the dorsal edge of the maxillary process.	Maxillary/Mandibular	Trouble
**11 (28)**	Point at the nasal aperture representing the intersection of the lateral nasal process and the maxillary process.	Maxillary/Mandibular	
**12 (29)**	The most rostro-ventral intersection between the medial nasal process and the maxillary process. Between E10.5 and E11.5, it shows the growth of the medial nasal process as a contributor to the labial margin.	Nasal	
**13 (30)**	The rostro-caudal most extension of the lateral nasal prominence, illustrating the rostral growth of the lateral nasal process between E10.5 and E12.5.	Nasal	Trouble
**14 (31)**	Point representing the middle of the medial side of the nasal aperture as a lateral extent of the medial nasal process.	Nasal	
**15 (32)**	The dorsal most point of the nasal aperture representing the rostro-dorsal extreme of the border between the medial and lateral nasal processes.	Nasal	
**16 (33)**	The corner of the developing mouth, found at the most caudo-lateral point on the rostral border of the maxillary and mandibular processes.	Maxillary/Mandibular	
**17 (34)**	The lateral extent of the center of the maxillary process as it exists in E10.5 and E11.5.	Maxillary/Mandibular	Trouble
**18 (35)**	The intersection of the buldge of the trigeminal ganglion and the pontine flexure of the developing brain.	Non-Facial	Trouble
**20 (37)**	The medial, rostral, dorsal corner of the developing mandibular process.	Maxillary/Mandibular	
**21 (38)**	The ventral caudal most point on the bulge of the growing forebrain, as noted from the lateral perspective.	Non-Facial	Trouble
**22**	The most caudal midline point on back of the head, just ventral to the midbrain.	Non-Facial	

**Figure 2:**
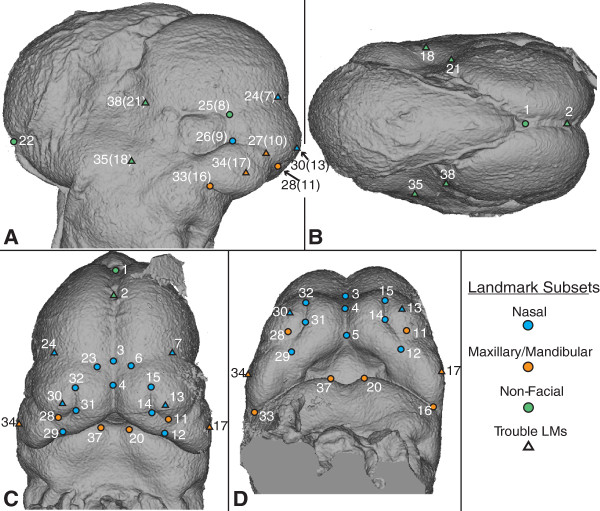
**Landmarks defined on the craniofacial ectoderm.** Landmark locations identified on the **A)** right lateral, **B)** dorsal, **C)** rostral, and **D)** rostro-ventral views of an E11.5 reference specimen, including the identification of the landmark subsets removed from ontogenetic regression analyses. Landmarks identified on 3D surfaces of all three ages are available in Additional file 5.

Simplified biological definitions for all landmarks as well as landmark subset definitions. Full biological and practical definitions are provided in Additional file 1. Definitions of anatomical directions used here are found in Figure 1. Identifications of these landmarks on specimens are found in Figure 2 and in Additional file 5.

Landmark homology is widely recognized as a critical feature of sparse landmark based morphometrics, although the definition of homology may shift depending on research question and sample [29,30]. Previous landmark based studies of mouse embryos between E10.5 and E12.5 are typically related to questions of growth and development of facial prominences and the structures derived from them [5,6,23-25]. Therefore, our landmark set, influenced greatly by this previous work, is composed of points at the borders between or at the extreme edges of prominences and/or other craniofacial features. Realizing that the specific cells found at the edges of a facial prominence may shift as it grows outward (discussed in [30]), we define homology based on the extent of the cellular populations whose proliferation and differentiation serve to modify the form of a given prominence and its derivative craniofacial features.

For example, point 9/26 is placed in a position that is near the ventral/rostral corner of the eye of E10.5 specimens (Figure 1). Based on this geometric relationship with the eye, this landmark might also be placed at the corner of the eye on E12.5 specimens. However, because our main questions are about the growth and relationship between the facial prominences, it is more important that landmark 9/26 continue to represent the caudal end of the border between the lateral nasal and maxillary processes at the developing lacrimal duct. It has been shown that the valley between the second and third whisker rows, counting from the dorsal aspect of the nose, represents the border between tissues derived from these cell populations [31,32]. Therefore, the E12.5 version of this point was defined at the posterior extreme of this valley between the whisker rows, found halfway up the anterior border of the eye (Figure 1). Similarly, we chose age specific locations for each landmark, as defined within the practical definitions, to represent homologous biological features, as defined within the biological definitions.

Even if landmarks represent relevant biological features well, landmarks cannot be used to measure subtle variation in craniofacial form if there is a large amount of variation in landmark coordinate identification. In order to test the variation in landmark placement for our landmark set (Table 1, Figure 2), we measured the intra- and inter-observer error associated with each landmark. After identifying some landmarks with relatively high error, we tested how removing groups of landmarks from our set influences the results of a simple ontogenetic analysis. This latter question addresses the issue of how the composition of the landmark set influences the extent to which the set captures and represents, overall, the shape of the face.

## Results

### Intraobserver error

**Figure 3:**
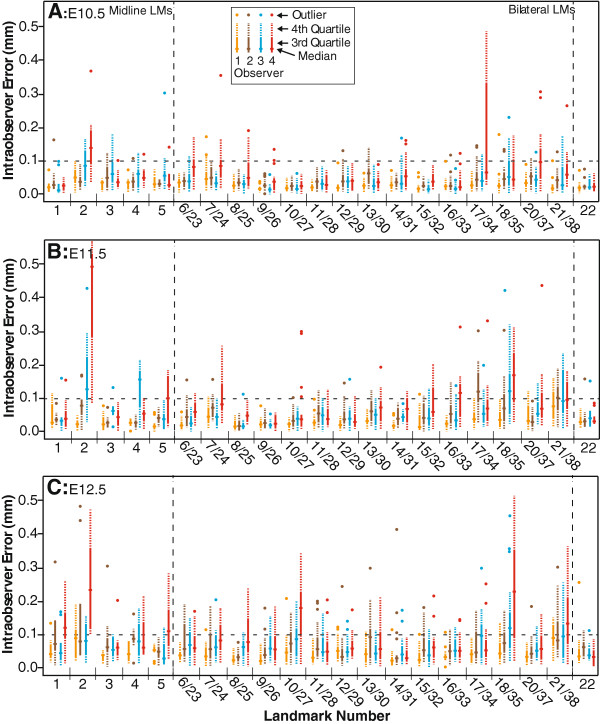
**Intraobserver landmark placement error.** Intraobserver error of each landmark (LM) for each observer at E10.5 **(A)**, E11.5 **(B)**, and E12.5 **(C)**, calculated as the euclidian distance between the landmark coordinates recorded in the two landmark placement trials. The calculations are combined for left and right versions of bilateral landmarks. The reference value of 0.1 mm discussed in the paper is shown as a dotted horizontal line. Observers 1, 2, and 3 are experienced with landmarking, while observer 4 is a first time landmarker.

Intraobserver error, the variation in placement of a given landmark from trial to trial by the same person, was measured as the Euclidian distance between the identified coordinates of a landmark during two landmark placement trails. After determining that left and right versions of bilateral landmarks showed similar patterns of intraobserver error, calculations of intraobserver error for bilateral landmarks include values from both sides. The median intraobserver error for landmarks taken by the more experienced landmarkers on E10.5 specimens (Figure 3A) tend to be well below 0.1 mm, usually closer to 0.05 mm, with the less experienced landmarker showing higher median and variance of error overall. While the more experienced landmarkers display low landmark placement error, the less experienced landmarker displays high median intraobserver error for landmark 2 and high variance for 17/34.For E11.5 embryos (Figure 3B), the median intraobserver error for the more experienced landmarkers is still well under 0.1 mm for most landmarks, although there are some landmarks that display higher error. Looking at the more experienced landmarkers, point 2 shows the highest median value, while point 4 is also high for one experienced observer. Of the lateral points, 17/34, 18/35, and 21/38 median values are above 0.1 for at least one of the experienced landmarkers. The median intraobserver errors are higher for the E12.5 embryos then the other two ages (Figure 3C), although this might be expected given that the overall dimensions of the head have increased substantially since E10.5. For E12.5, landmark 21/38 consistently displays the highest level of error for the experienced landmarkers, while the points 17/34, 18/35, and 13/30 display median close to 0.1 mm for at least one experienced landmarker.

### Interobserver error

**Figure 4:**
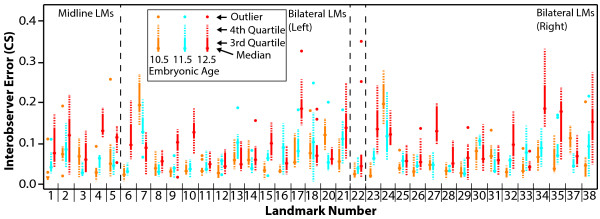
**Interobserver landmark placement error.** Interobserver error for each landmark (LM) at E10.5 (orange), E11.5 (blue), and E12.5 (red), calculated as the centroid size (CS) of the mean landmark coordinates of the three experienced landmarkers. Centroid size was calculated separately for the left and right side of bilateral landmarks.

Interobserver error was calculated for every landmark of every specimen as centroid size of the average landmark locations chosen by the three experienced landmarkers (average between trials 1 and 2). Although centroid size is not directly interpretable as a Euclidian distance, centroid size values are comparable between ages in our analysis (Figure 4).

Overall, the median and variance of interobserver error is lower for E10.5 and E11.5 data than for E12.5 data. Landmarks that were identified as having relatively high intraobserver error across ages, including 2, 13/30, 17/34, 18/35, 21/38 are among the landmarks with higher mean interobserver error at E10.5 and E11.5. However, the landmark that displays the most interobserver error at E10.5 and E11.5 is 7/24, which showed relatively low intraobserver error. At E12.5, landmark 17/34, 18/35 and 21/38 display he highest median interobserver error, although 18/35 seems to be much more problematic on the right side than the left.

### Significance test

A two-way analysis of variance (ANOVA) was carried out for each embryonic age on the distance between each observer’s landmark placement and mean landmark location with observer, specimen, and landmark identity as factors (Table 2). This was done in order determine the significance of the association of each of these factors with variation in landmark placement. Across all three embryonic days, there is a highly significant effect of observer and landmark on the strength of variation in landmark coordinates from the mean coordinates for each specimen. There is a highly significant effect of specimen identity on this coordinate variation at E10.5 and a significant effect at E11.5, indicating that some specimens tend to display higher variation in landmark placement between observers at the earlier ages.

**Table 2:** Results of two-way ANOVA for association between relevant factors and landmark coordinate variation

	**E10.5**			**E11.5**			**E12.5**		
	**DF**	**Sum Sq**	**p-value**	**DF**	**Sum Sq**	**p-value**	**DF**	**Sum Sq**	**p-value**
Specimen	9	0.0406	**4.6E-08**	8	0.0179	0.019	9	0.0340	0.1097
Landmark	3	0.0282	**6.9E-08**	3	0.0874	**<2.2E-16**	3	0.5048	**<2.2E-16**
Observer	35	0.5857	**<2.2E-16**	35	0.8070	**<2.2E-16**	35	3.1895	**<2.2E-16**
Residual	1392	1.0711		1249	1.2124		1392	3.2834	

Two-way ANOVA results for the association between Specimen, Landmark, and Observer factors and variation in identified landmark coordinates for the three embryonic ages. Results include degrees of freedom (DF), sum of squares (Sum Sq), and p-value, which is highlighted in bold if highly significant (<.001). Variation in landmark coordinates were calculated as Euclidian distances between a given landmark location and the mean location across observers for the same specimen.

### Ontogenetic analysis

**Figure 5:**
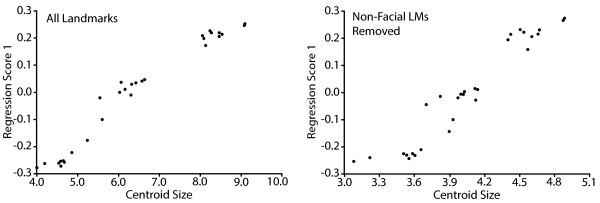
**Results of a regression of landmark coordinates on centroid size.** The association between centroid size and a summary score of the landmark coefficients associated with a multiple multivariate regression of landmark coordinates on centroid size (Regression Score 1). This association is illustrated for regressions when all landmarks (LMs) were included (left) and when non-facial landmarks were removed (right), which represent the high end and low end of linearity for the regressions in our analyses.

**Figure 6:**
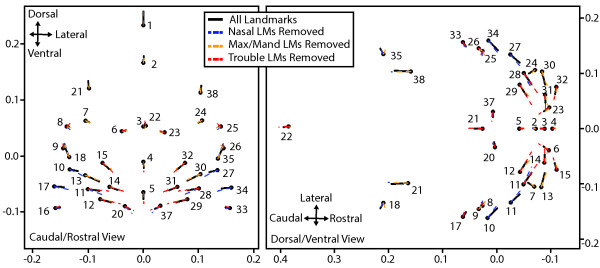
**Comparison of ontogenetic shape change vectors for landmark subsets.** Comparison of ontogenetic shape change vectors for regressions of landmark coordinates on centroid size for all landmarks (black), when nasal landmarks (LMs) were removed (blue), maxillary/mandibular removed (orange), and trouble landmarks removed (red). The vectors are based on the regression coefficients for each axis of a given landmark and all plotted relative to the mean procrustes coordinates of that landmark calculated when including all landmarks. The values of all axes are procrustes coordinates.

We identified a list of trouble landmarks, based on their high median intra- or inter-observer error values, for which we did not believe a clarification of landmark definition would necessarily reduce error (see Discussion). In order to determine how removing these trouble landmarks or 3 other morphologically defined landmark groups from our sample would modify the results of a simple ontogenetic analysis, we completed separate regressions of landmark coordinates on centroid size for five subsets of landmarks within MorphoJ (Table 1, Figure 2).The association between a summary regression score and centroid size is roughly linear for each of the five regressions completed (Figure 5). The proportion of the total variation for which the regression including all landmarks accounts is approximately 82.5%, while it is 78.9% when the trouble landmarks are removed, 83.8% with nasal landmarks removed, 81.1% with maxillary/mandibular landmarks removed, and 73.3% with non-facial landmarks removed. Based on a subjective comparison of the shape vectors associated with each regression, the removal of any of these subsets of landmarks does not grossly change the nature of the allometric shape change of a given landmark during the developmental period under study (Figure 6). As the embryo develops between E10.5 and E12.5, the bilateral landmarks of the face become relatively more rostral and closer together. The facial landmarks closer to the midline also become relatively more ventral by E12.5. The landmarks found away from the face are relatively more caudal and dorsal in the older specimens.

## Discussion

### Intra- and inter-observer error

Bookstein [28] defined a commonly used landmark classification system based on the features used to determine landmark location. Type 1 landmarks are defined as the location of a discrete anatomical structure, independent of other anatomical features. Several of the landmarks in our set (Figure 2) are Type 1, including 8/25 that is found at the intersection of the eye, forebrain, and lateral nasal prominence. Type 2 landmarks are extreme points along curves or grooves and represent the majority of landmarks defined on the surfaces of embryos for this and previously defined sets (eg. [6,25]). Type 3 landmarks, those defined with respect to relatively distant anatomical structures, do not necessarily share biological meaning and homology across specimens. They are considered mathematically deficient, because their locations are partially dependent on the locations of other anatomical structures [28,33]. However, because Type 1 and Type 2 landmarks cannot comprehensively represent the shape of the early facial prominences [3], we defined a few landmarks on the extreme of craniofacial bulges in order to improve our coverage of the anatomical features of interest. Because the face develops quickly between E10.5 and E12.5 (Figure 1), biologically homologous landmarks may switch between landmark types across this period of development. For example, landmark 17/34 is the extreme lateral edge of the maxillary prominence at E10.5 and E11.5 (type 3), but is defined based on location between whisker rows at E12.5 (type 2). Even if a landmark does not change type, the error associated with its identification may change across this developmental period.

Our landmark set was compiled with the goals of 1) creating an explicit homologous association between each landmark and a biological feature across E10.5-E12.5 and 2) serving as the basis for ontogenetic analyses that include datasets produced by multiple observers. Therefore, we have attempted to choose landmarks that represent relevant biological features well and which can be repeatably placed between trials and by multiple observers. However, we acknowledge that a given landmark may not necessarily be more biologically relevant or repeatable than other nearby locations, particularly if the landmark is defined along a curve or curved surface (i.e. type 2 and 3 landmarks). We have endeavored to transparently report our biological interpretations of landmarks and the repeatability of landmark placement in order to allow other researchers to judge the usefulness of this landmark set for their own studies.

None of the landmarks identified as having high intra- or inter-observer error in this study are Type 1 landmarks. Instead, they tend to be Type 2 landmarks along curves with wide inflection (eg 2, 7/24, 18/35) or Type 3 landmarks on the extreme extents of bulges (eg. 17/34). After reviewing the landmarks identified as having high error (the subset of trouble landmarks) we are confident that we can clarify the definitions of 10/27 and 13/30 in order to reduce landmark placement error, based on noted systematic differences in placement between landmarkers. However, we suggest that 2, 7/24, 17/34, 18/35, and 21/38 be removed from our standardized landmark set for E10.5-E12.5 mice. This does not mean that these landmarks are necessarily inappropriate for a specific embryonic age or as an additional landmark for studies that include only one observer. For instance, landmark 7/24 displays high interobserver error and relatively low intraobserver error for the experienced observers, suggesting that observers developed their own stable (although different) interpretations of the landmark. Similarly, the median intraobserver error across the three experienced landmarkers increases for landmark 21/38 from E10.5 to E12.5 as the forebrain expands, suggesting that the landmark may be appropriate for use at E10.5 but not at E12.5.

After removing trouble landmarks from our landmark set, and assuming minimal modification of craniofacial shape during preparation and imaging of specimens [24], median intraobserver error of our experienced landmarkers is below 0.1 mm. In the case of E10.5 specimens, it tends to be below 0.05 mm. Therefore, the application of this landmark set by an experienced landmarker should allow the capture of differences in facial shape between groups of specimens that are significantly larger than 0.1 mm. For comparative purposes, the width of the ridge lateral to the nasal aperture is 0.13 mm at E10.5, while the width of the nasal aperture measured from the rostro-ventral extent of the lateral and medial ridges is 0.14 mm at E12.5. The significantly higher intraobserver error noted for the first time landmarker highlights the need for landmark training and experience in order to reduce error to acceptable levels.

One of the major reasons to design this standard set of landmarks was to allow landmarks taken by multiple observers to be combined in comparative analyses. Unfortunately, the interobserver error noted for many landmarks indicates significant differences in the interpretation of landmark definitions even though we provided precise biological and practical landmark definitions, examples of landmarks identified on a 3D craniofacial surface for each age, illustrations of standard head orientation, and face to face discussion. Measures of interobserver error can illustrate the need for further clarification of certain landmark definitions or the removal of a landmark from analysis entirely. Alternatively, assuming that there is high interobserver and low intraobserver error, it may be possible to remove typical differences in interobserver landmark placement via regression of landmark coordinate values on observer identity. While completely removing interobserver landmark error may not be possible, comparative error studies like this are highly recommended in order to identify and address problematic landmarks as a way to reduce the level of landmark placement error included within subsequent analyses.

The variation in landmark placement, measured as the distance between an observer’s landmark coordinates and mean landmark coordinates, is strongly associated with the factors of landmark and observer identity at E10.5, E11.5, and E12.5, according to our two-way ANOVA analysis. Specimen identity is associated with landmark variation at E10.5 and E11.5, although with reduced significance at E11.5 (Table 2). The significant effect of observer identity on landmark coordinate variation indicates that significant differences in landmark placement exist between observers for at least some of the landmarks. The significant effect of landmark identity suggests that this interobserver variation in landmark placement is higher for some landmarks than others. Although significant at all three embryonic days, the sum of squares values for both observer and landmark increase from E10.5 through E12.5, which matches the observation that mean inter- and intra-observer error values tend to increase from E10.5 to E12.5 (Figures 3 and 4). The significant effect of specimen identity in the earlier ages does not suggest that interobserver error is higher for younger specimens. It merely suggests that the level of interobserver error varies between specimens at the younger ages. This difference in landmark placement error may be associated with larger relative differences in morphology between specimens at slightly different stages of development within the younger age categories. It may also be due to relatively more significant fixation based changes in morphology within some younger specimens.

### Interpreting ontogenetic analyses

Another major reason to define this set of landmarks was as a basis for ontogenetic analyses of craniofacial shape in mice between E10.5 and E12.5. Therefore, we carried out a series of linear regressions between size and shape in order to determine how the selection of landmarks might influence the results and interpretation of this type of analysis. Omitting major groups of landmarks from these regressions did not substantially modify the nature of the craniofacial shape change associated with an increase in centroid size between E10.5 and E12.5, suggesting that the specific landmarks selected should not influence interpretation of shape change across this ontogenetic period.

However, removing the non-facial or trouble landmarks from the dataset reduced the linearity between landmark coordinate regression coefficients and centroid size, as well as the total landmark coordinate variation explained by the regression on centroid size. In our regressions, centroid size is a proxy for developmental age. Between E10.5 and E12.5, the growth of the facial prominences outward is not as pronounced as the growth and definition of the various regions of the brain [34] (Figure 1). The lower explanatory power of the regression when non-facial landmarks were removed suggests that including these landmarks improves the usefulness of centroid size as a proxy for developmental age, because it is linked to the overall change in craniofacial size over this developmental period. The intermediate reduction in explanatory power for the regression when the trouble landmarks were removed is likely based on the fact that a high proportion of the trouble landmarks are non-facial landmarks.

We recommend that landmarks across the head be included in ontogenetic analyses in order to better estimate centroid size as a measure of developmental age, but also to provide a broader anatomical context within which to interpret morphological changes of the face or another specific region of interest. If possible, using a measure of developmental age that is independent of the landmarks under analysis, such as tail somite number, may be preferable [6].

### Future directions

Great progress has been made in the automation of craniofacial surface quantification and comparison ([35-41]), which might make issues of intra- and inter-observer landmark placement error irrelevant. However, despite significant effort, an adequate automatic method has not yet been developed that allows for the quantification of subtle craniofacial variation in young embryos. This is partially due to difficulty in producing standardized and complete ectodermal surfaces from μCT images, as well as the fact that craniofacial features change very quickly during embryonic development to the point where a great deal of variation exists within a sample of specimens from a single embryonic day. The human eye remains, unfortunately, superior to computational methods in locating homologous features on incomplete surfaces and across gradations of developmental age. The authors are involved in work to automate embryonic landmark placement, but must continue to rely on manual placement of landmark sets like this for the time being.

## Conclusion

Standardization of landmarking protocols is a necessary first step, but not sufficient for phenotypic data to become phenomic data. It must also be shown that the landmark coordinates identified using the standard protocol are sufficiently comparable between landmark trials and between observers. We developed and tested a set of ectodermal surface landmarks for the measurement of craniofacial morphology of mice between E10.5, E11.5, and E12.5. Our package of landmark definitions, illustrations, and examples were developed in an effort to reduce variation in placement for all landmarks in our set. Measurements of intra- and inter-observer error for four observers reinforce the idea that landmark coordinates identified from one landmarker will have less variability than landmarks taken by multiple observers. Intraobserver error comparisons also highlight the need for landmarkers to be provided significant training and supervision before the incorporation of their landmark sets into larger meta-analyses including multiple observers. However, we are confident that this standard landmark set, once landmarks with higher error are removed, can serve as a basis for the comparison of landmarks individually collected by experienced landmarkers for different mouse strains and disease models. Finally, our results suggest that the nature of craniofacial shape changes identified with simple ontogenetic analyses are robust to the particular choice of landmarks that are included in those analyses. By using these landmarks within a variety of future studies, perhaps as part of larger study or age specific landmark sets, we plan to build a dataset for the comparison of facial morphogenesis across many mouse populations in order to help identify the developmental bases for phenotypic variation in the craniofacial complex.

## Methods

### Landmark definitions and data collection

This study was performed using a sample of 10 E10.5, 9 E11.5, and 10 E12.5 CT images heads from mice of various backgrounds that were collected for other studies. None of these mice display gross dysmorphology during the early embryonic period. Care and use of mice for this study were in compliance with relevant animal welfare guidelines approved by the University of Colorado - Denver and the University of Calgary. Fixation in 4%PFA/5% Gluteraldehyde was carried out according to protocols designed to minimize the level of desiccation and craniofacial shape change in these embryonic specimens [24]. All μCT images chosen for this study were produced with a Scanco μ35 at the University of Calgary with 45 kV/177uA for images of 0.007 mm (E10.5 and E11.5) or 0.012 mm (E12.5) voxel size.

All landmarks in our landmark set (Table 1; Figure 2) were given a biological definition representing a biological interpretation of that landmark across all three ages of interest. A practical landmark was defined for each landmark at each age in order to guide the placement of the landmark so that its location at each age would match the biological definition. Because some of these landmarks are defined as the extremes of curved surfaces, the practical definitions may include instruction on the orientation of the surface image that a landmark should be taken from. 2D lateral images of an embryonic head from each age were produced to define a standard orientation for landmarking. Terms of anatomical direction used within the definitions are in reference to a mouse head in standard adult position and may not match the directions as defined by the embryonic body. Full landmark definitions and orientation images are available in Additional files 1, 2, 3, 4 and 5. Last, each landmark was placed on the 3D surface of a reference specimen of each embryonic age as an example to be referenced during the process of data collection. Because of the relatively large file sizes involved, these surface files and associated landmark coordinates are available upon request to the authors.

After a brief orientation and access to all landmark reference materials, four observers completed two landmark trials within MeshLab [42] on minimum-threshold based ectodermal surfaces produced from μCT images of each specimen. Trials 1 and 2 were completed at least one week apart in order to reduce the influence of memory on landmark placement during the second landmarking trial. The first two observers (1,2), who had experience collecting landmarks from CT images of mice, collaborated to develop the landmark set definitions, the third (3) was also an experienced mouse landmarker, and the fourth (4) was a first time landmarker (numbers as defined in Figure 3.

### Landmark error analysis

Intraobserver landmark placement error was calculated for each landmarker (1-4) as Euclidian distances (mm) between the location of a landmark taken during landmark trials 1 and 2. Boxplots were used to visualize the median and variation of intralandmark error for each landmarker (Figure 3). After determining that the left and right versions of bilateral landmarks show similar levels of intraobserver error, we decide to combine these values for both sides when calculating intraobserver error for the bilateral landmarks.

Inter-observer landmark placement error was first calculated as the centroid size of the mean landmark coordinates defined by each observer for each landmark (mean of trials 1 and 2). Because of the high intraobserver error noted for the less experienced observer, our interobserver error and all further analyses focus on the data from the three more experienced observers.

The mean landmark coordinates for a given specimen were calculated as the average of the mean landmark coordinates calculated from the two trials of each observer. Variation in landmark coordinates were calculated as the Euclidian distance between the coordinates collected by a given observer on a given specimen and the mean coordinates for that specimen. A two-factor analysis of variance (ANOVA) was completed for each embryonic age to test whether specimen, landmark, or observer identity are significantly associated with this variation in landmark coordinates.

### Ontogenetic analysis

Regressions of procrustes coordinates against centroid size, a better proxy for developmental age than embryonic day, were performed in MorphoJ [43] to determine the strength and nature of ontogenetic change in craniofacial shape from E10.5 to E12.5. This regression was performed for the whole dataset and a dataset from which trouble landmarks with high intra- or inter-observer error were removed (Table 1; Figure 2). To further investigate how the use of different subsets of landmarks might influence the results of an ontogenetic analysis, regressions of procrustes coordinates against centroid size were also calculated for the dataset after removing nasal, maxillary/mandibular, or non-facial landmarks (Table 1; Figure 2). A summary regression score representing the regression coefficients of each specimen [44] was plotted against centroid size in order to visualize the strength and linearity of this association between craniofacial shape and centroid size. An estimate of the proportion of total variation for which a regression accounts, an analogue of an R-squared value, serves as an informal measure of the strength of this association.

The vectors of landmark change associated with centroid size provide a summary of the nature of the ontogenetic changes in craniofacial shape between E10.5 and E12.5. These vectors are calculated as regression coefficients of each axis of each landmark added to the mean procrustes coordinate of the landmark along each axis. A figure displaying the relative ontogenetic vectors associated with four of the landmark subsets required the use three landmarks to define parallel axes for procrustes superimposition of each subset as the basis for each regression against centroid size (these three landmarks were not found in the fifth subset of landmarks). The resulting coefficient vectors were plotted relative to the mean shape of the dataset including all landmarks for the purpose of visualization in R [45].

### Availability of supporting data

The full landmark definitions, the original embryo orientation images, and an image illustrating the placement of the landmarks at all three embryonic days are available as Additional files 1, 2, 3, 4 and 5. Because of the size of the 3D surface meshes, these meshes and associated landmark coordinates for reference specimens at E10.5, E11.5, and E12.5 are available upon request to the authors.

## Abbreviations

μCT: Micro computed tomography; E: Embryonic day (followed by the number of days).

## Competing interests

The authors declare that they have no competing interests.

## Authors’ contributions

All authors participated in the design of the study. CJP and RG defined the landmarks and carried out data collection. CJP completed analysis of the landmark coordinate data and wrote the initial draft of the manuscript. All authors contributed to and approve the final manuscript.

## Supplementary Material

Additional file 1 **Full biological and practical landmark definitions.** The practical definitions, which can differ between embryonic ages are intended to guide landmark placement so that landmarks are placed homologously across ages according to the biological definitions. Click here for file

Additional file 2 **Standard orientation for E10.5 embryos.** An image defining standard lateral orientation of E10.5 embryonic specimens for landmark placement. Click here for file

Additional file 3 **Standard orientation for E11.5 embryos.** An image defining standard lateral orientation of E11.5 embryonic specimens for landmark placement. Click here for file

Additional file 4 **Standard orientation for E12.5 embryos.** An image defining standard lateral orientation of E12.5 embryonic specimens for landmark placement. Click here for file

Additional file 5 **Landmark locations identified on embryos of all three ages under study.** These landmarks are defined in Table 1 and Additional file 1. These landmarks are colored by groups on an E11.5 specimen within Figure 2. Click here for file
