# Supplementary figures and images for: Surface landmark quantification of embryonic mouse craniofacial morphogenesis
Source: BMC Dev Biol. 2014 Jul 24;14:31. doi: 10.1186/1471-213X-14-31 (PMC4222779; doi:10.1186/1471-213X-14-31)

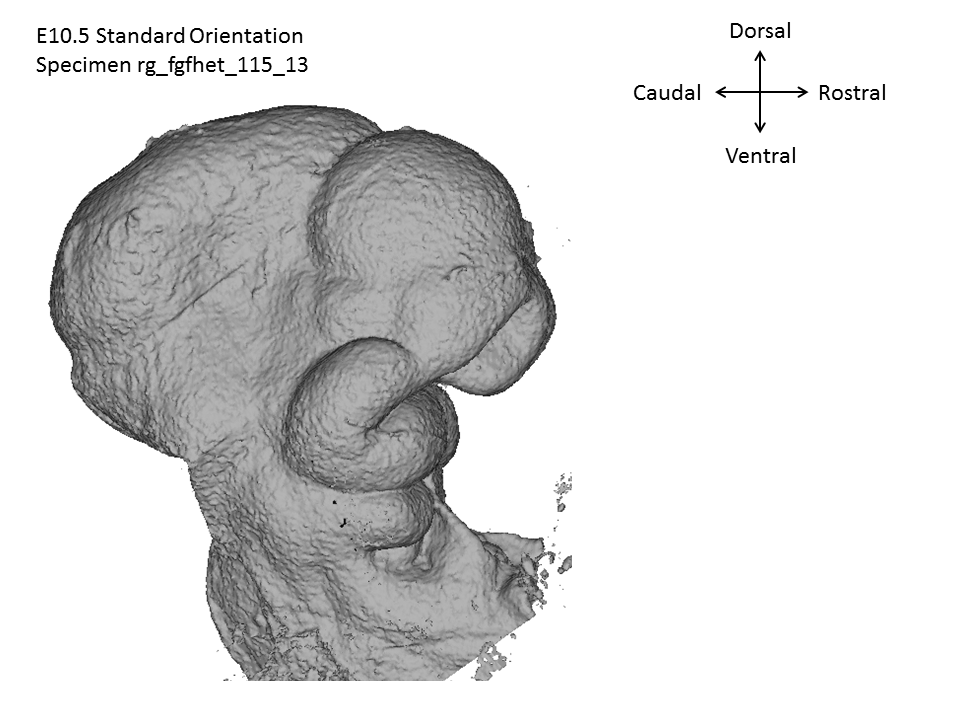

Supplement: Additional file 2 — Standard orientation for E10.5 embryos. An image defining standard lateral orientation of E10.5 embryonic specimens for landmark placement. [file 1471-213X-14-31-S2.tiff]

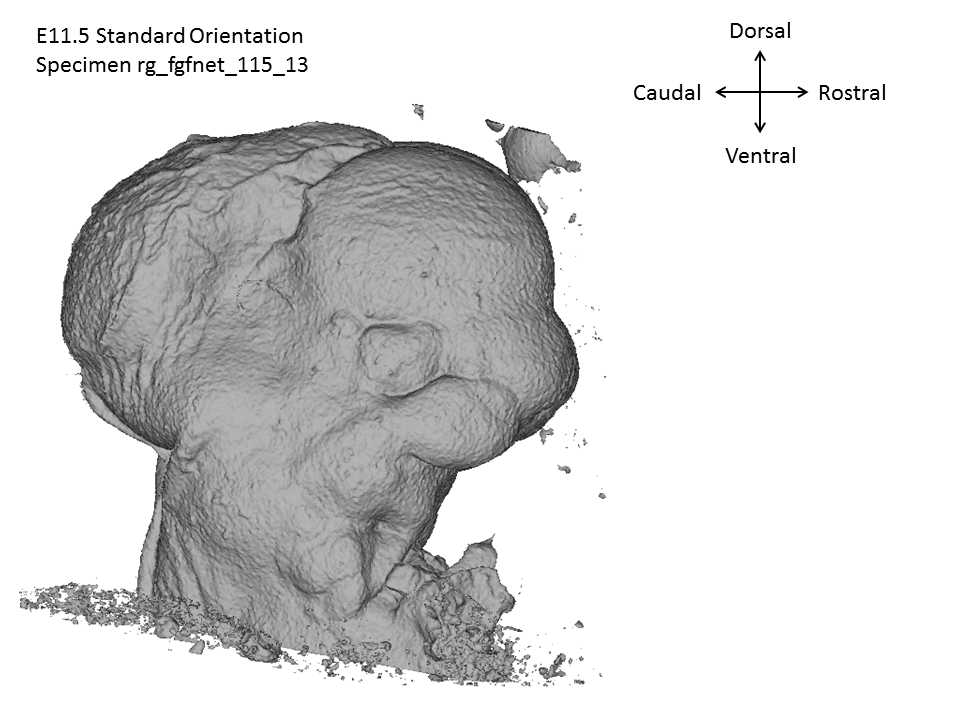

Supplement: Additional file 3 — Standard orientation for E11.5 embryos. An image defining standard lateral orientation of E11.5 embryonic specimens for landmark placement. [file 1471-213X-14-31-S3.tiff]

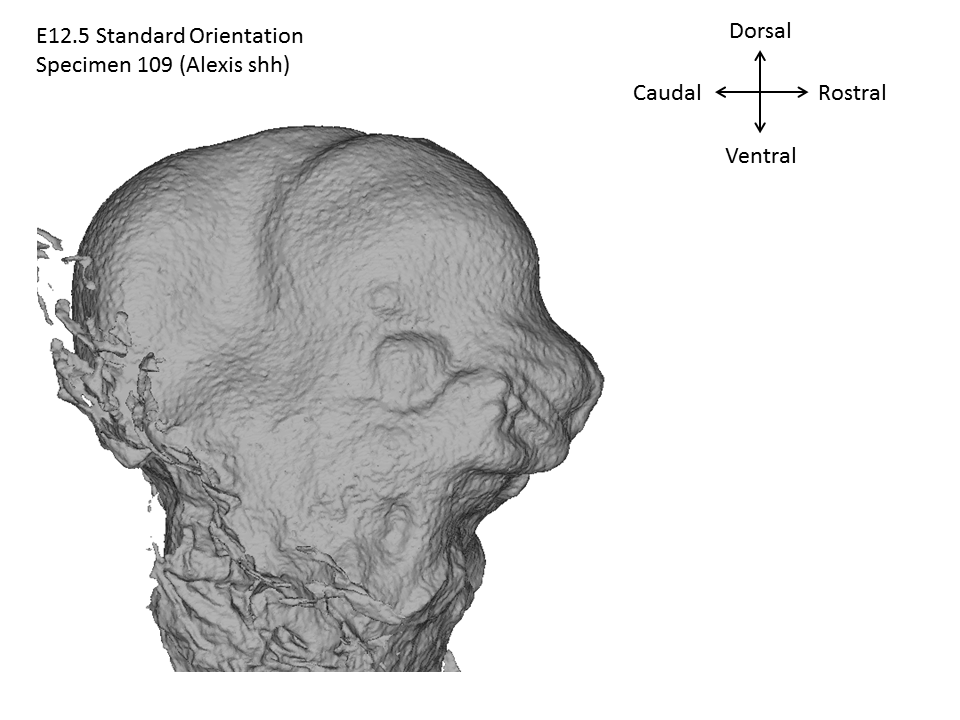

Supplement: Additional file 4 — Standard orientation for E12.5 embryos. An image defining standard lateral orientation of E12.5 embryonic specimens for landmark placement. [file 1471-213X-14-31-S4.tiff]

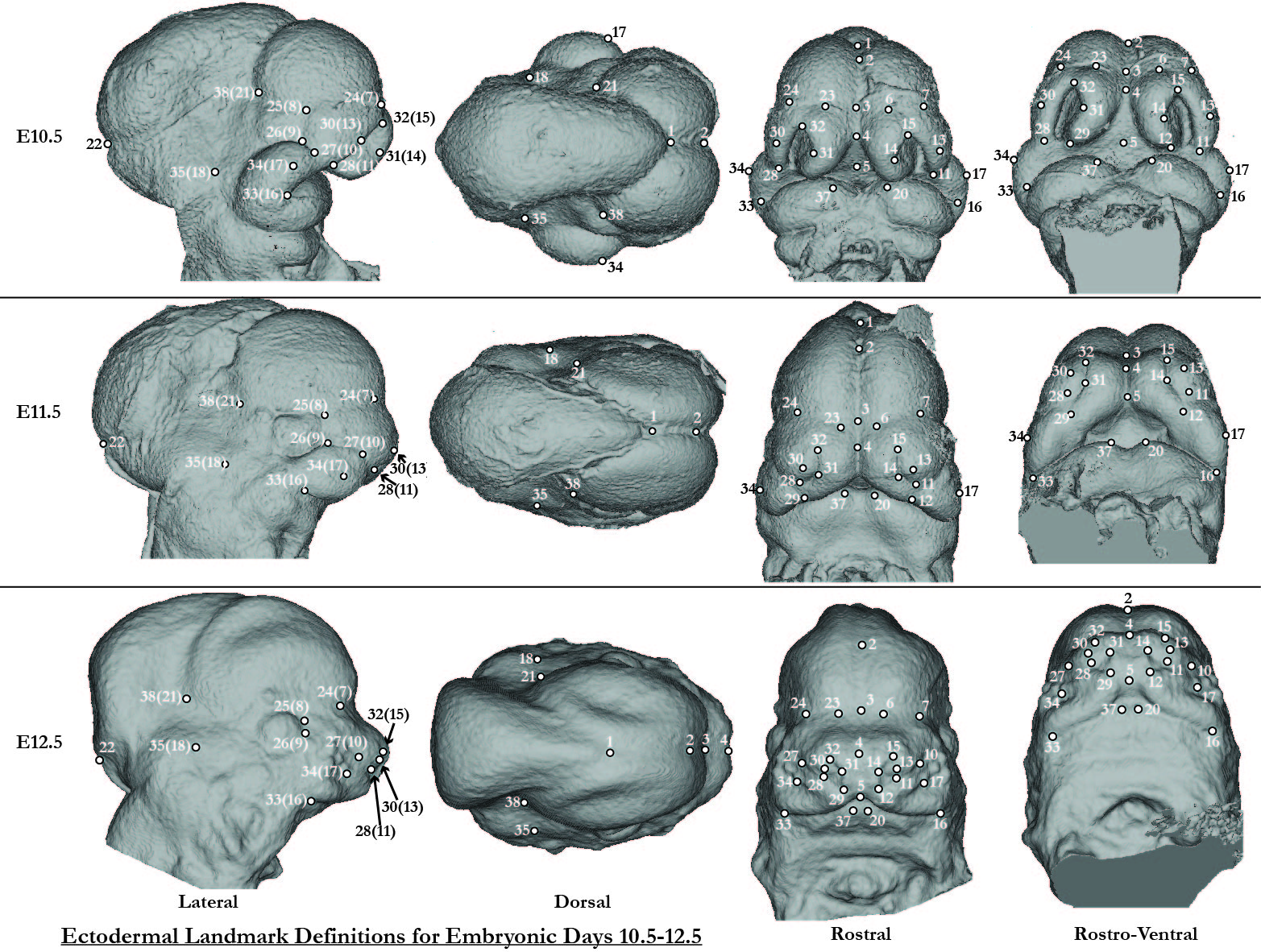

Supplement: Additional file 5 — Landmark locations identified on embryos of all three ages under study. These landmarks are defined in Table 1 and Additional file 1. These landmarks are colored by groups on an E11.5 specimen within Figure 2. [file 1471-213X-14-31-S5.jpeg]
